# Remarkable response to pembrolizumab in PD-L1 overexpressing (≥ 50%) NSCLC and extracranial abscopal effect induced by brain radiotherapy: a case report

**DOI:** 10.3389/fonc.2025.1652515

**Published:** 2025-12-12

**Authors:** Yong Xia, Hongfan Zhu, Shaoqing Huang, Xinhong Guan, Xiuxiu Chen, Qian Zhang, Liu Meng, Hui Xue, Haiyan Xiang, Shenglan Lai, Jinxing Lou

**Affiliations:** 1 Department of Biotherapy, Shanghai University Affiliated Mengchao Cancer Hospital, Shanghai, China; 2 Department of Thoracic Surgery, Ningbo No.2 Hospital, Ningbo, China

**Keywords:** non-small cell lung cancer, programmed cell death ligand 1, abscopal effect, radiotherapy, immunotherapy

## Abstract

**Background:**

Lung cancer remains the most prevalent malignant neoplasm worldwide, with adenocarcinoma being among its most frequent subtypes. The brain is a common metastatic site in patients with lung adenocarcinoma, often associated with poor prognosis, and brain radiotherapy is the standard recommended treatment. However, the occurrence of an extracranial abscopal effect following brain-directed radiotherapy is rare due to the brain’s unique immune microenvironment.

**Case description:**

We present the case of a 64-year-old Asian male who was admitted with complaints of “right-sided hemiplegia, reduced muscle strength, impaired ambulation, headache, projectile vomiting, and fatigue persisting for five days”. Magnetic Resonance Imaging (MRI) of the brain revealed multiple space-occupying lesions in the bilateral frontal lobes, left cerebellum, and posterior horn of the right lateral ventricle. The patient underwent palliative brain radiotherapy (targeted to the left frontal lobe and right lateral ventricle posterior horn), after which a significant extracranial abscopal effect was observed even before systemic therapy initiation, accompanied by significant improvement in neurological symptoms. Contrast-enhanced Computed Tomography (CT) of the chest demonstrated multiple space-occupying lesions in both lungs (more prominent in the lower lobe of the left lung), along with metastases involving the bilateral mediastinum, left hilar region, and bilateral supraclavicular lymph nodes. Histopathological evaluation of a biopsy obtained from the right supraclavicular lymph node, supported by morphological and immunohistochemical findings, confirmed metastatic lung adenocarcinoma with a PD-L1 tumor proportion score (TPS) ≥50%. Molecular profiling revealed a KRAS G12C mutation, while EGFR, ALK, and ROS1 alterations were absent. In accordance with NCCN guidelines, the patient received monotherapy immunotherapy with a PD-1 antibody. He achieved a sustained partial response both intracranially and extracranially for up to 24 months, with substantial improvement in quality of life.

**Conclusion:**

This case highlights that an extracranial abscopal effect can occur following brain radiotherapy alone in lung adenocarcinoma patients with brain metastases and PD-L1 TPS ≥50%. For such patients, the combination of palliative brain radiotherapy and PD-1 antibody therapy may represent a safe and effective therapeutic strategy.

## 1 Introduction

Non-small cell lung cancer (NSCLC) remains the leading cause of cancer-related mortality worldwide and poses a particular challenge in patients who develop brain metastases (BMs) (1, 2). Approximately 20%–60% of individuals with advanced NSCLC will eventually develop BMs, and 7%–10% of cases present with intracranial involvement at the time of initial diagnosis. Without treatment, brain metastases are associated with a dismal prognosis, often leading to death within 1–2 months (3). In recent years, advances in radiotherapy, surgery, molecularly targeted therapies, and immune checkpoint inhibitors (ICIs) have significantly expanded treatment options for lung adenocarcinoma with brain metastases. Among these, ICIs are particularly noteworthy because they can cross the blood–brain barrier and demonstrate antitumor activity within the central nervous system. Compared with palliative chemotherapy, pembrolizumab has been shown to prolong both progression-free survival (PFS) and overall survival (OS) in NSCLC patients with or without brain metastases who express programmed cell death ligand 1 (PD-L1) with tumor proportion score (TPS) ≥1%, achieving an intracranial response rate of 20%–30% (4, 5).

For patients with NSCLC who present with symptomatic brain metastases, timely and active local treatment is essential. When the number of brain lesions is ≤3, several options are available: (1) surgical resection, (2) stereotactic radiotherapy (SRT), or (3) SRT in combination with whole-brain radiotherapy (WBRT). For patients with >3 brain metastases, WBRT or SRT may be considered as appropriate strategies.

The abscopal effect describes a unique phenomenon in which local treatment, most commonly radiotherapy, exerts systemic antitumor activity. Beyond its direct cytotoxic effect on the irradiated lesions, radiotherapy may stimulate the immune system to mount an antitumor response against distant, untreated tumor sites. The occurrence of an extracranial abscopal effect following brain-directed radiotherapy alone is exceedingly rare, likely due to the specialized immune microenvironment of the brain (6, 7).

In the present study, we describe a rare case of lung adenocarcinoma with PD-L1 TPS ≥50% in which an extracranial abscopal effect was observed following brain radiotherapy alone, accompanied by a striking and durable response to subsequent pembrolizumab therapy.

## 2 Case report

On May 5, 2023, a 64-year-old Chinese male presented to the Biotherapy Center of Shanghai Mengchao Cancer Hospital with complaints of “right-sided hemiplegia, decreased muscle strength, inability to ambulate, headache, projectile vomiting, and fatigue for five days”. Positron emission tomography–computed tomography (PET-CT) revealed a malignant lesion in the lower lobe of the left lung, accompanied by multiple metastatic deposits in the bilateral lungs, left hilar and mediastinal lymph nodes, bilateral supraclavicular lymph nodes, as well as numerous intracranial and osseous metastases (**Supplementary Figure S1**). Given the extensive tumor burden, the patient’s initial prognosis was considered poor.

Furthermore, his medical history was notable for hepatocellular carcinoma, for which he underwent radical hepatectomy in May 2012, with postoperative pathology confirming the diagnosis. To date, no evidence of recurrence has been observed. The patient reported a long-term history of tobacco use (20 cigarettes daily for approximately 30 years) and alcohol consumption (beer, three times per week, ~400 mL per occasion). He denied a history of diabetes, hypertension, autoimmune disorders, or other chronic medical conditions.

**Figure 1:**
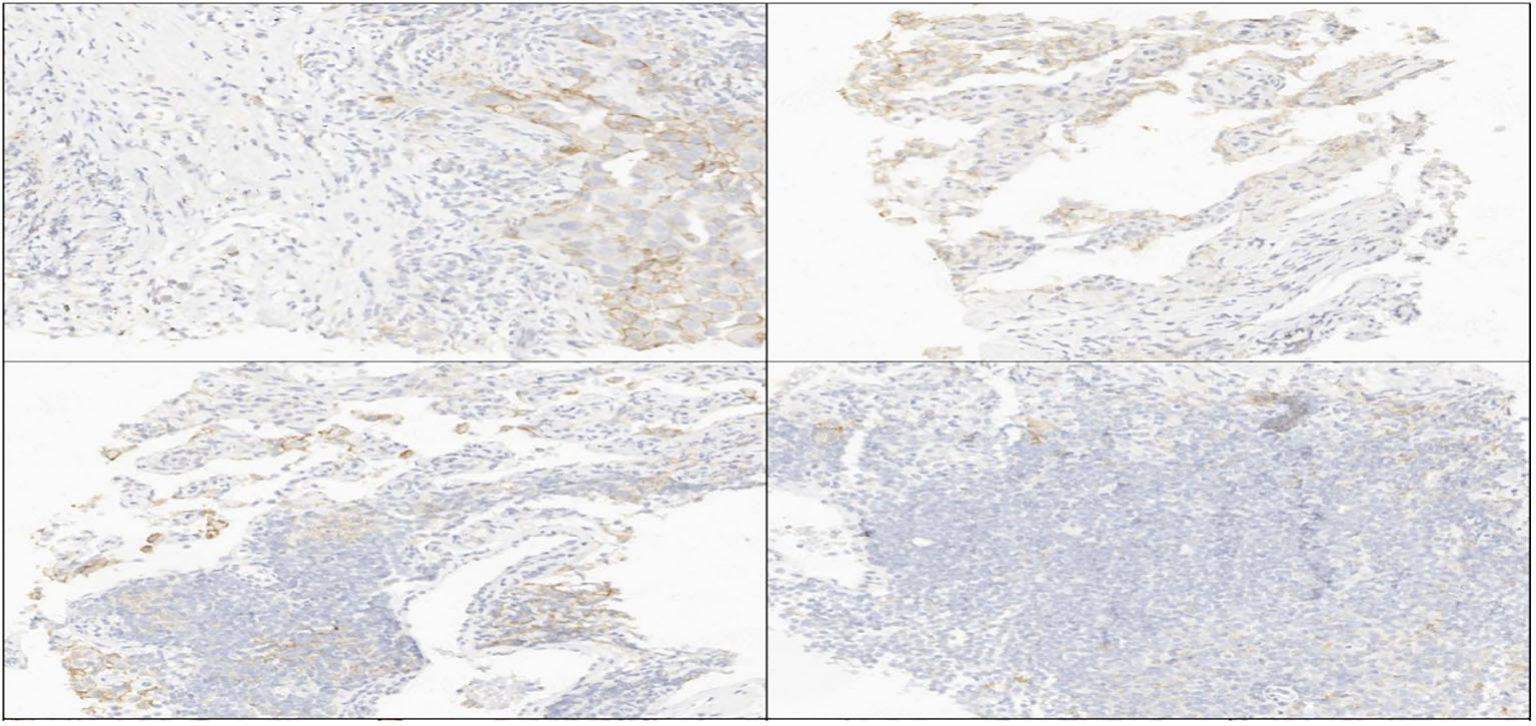
FFPE tissue photomicrograph from lymph node metastatic lesion stained for PD-L1 (TPS=60%) assessment. L1,programmed cell death receptor-ligand 1.

Following multidisciplinary consultation with the radiotherapy department, palliative radiotherapy was initiated to alleviate hemiplegia, targeting metastatic lesions in the left frontal lobe and the posterior horn of the right lateral ventricle (Intensity-Modulated Radiation Therapy [IMRT]: 2 Gy × 14 fractions). On May 18, 2023, a core needle biopsy of the right supraclavicular lymph node mass was performed. Histopathological evaluation confirmed metastatic lung adenocarcinoma. Immunohistochemical analysis demonstrated the following profile: CK7 (+), TTF-1 (+), Napsin A (+), P40 (–), MET (90%), CK20 (–), ALK (–), PAX-8 (–), WT-1 (–), Ki-67 (+, ~30%), and PD-L1 (TPS = 60%) (**Figure 1**).

**Figure 2:**
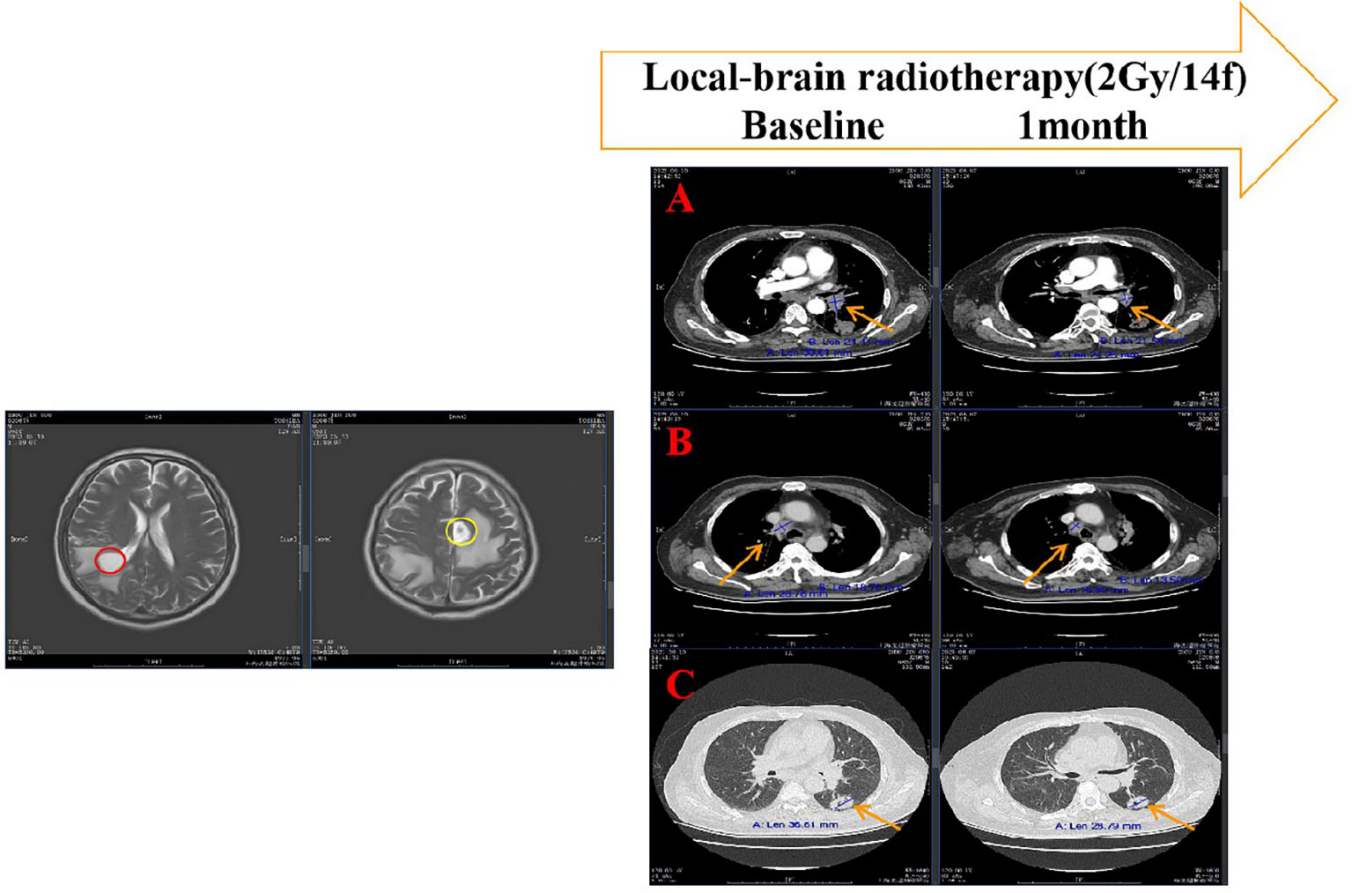
Extracranial abscopal effect induced brain radiotherapy. The right lateral ventricle posterior corner (red circle) and the left frontal lobe (yellow circle) metastasis are the gross tumor volume of radiotherapy.After brain radiotherapy, the left hilum of lung **(A)**, the right mediastinal lymph node **(B)** and the left lung lesion **(C)** were significantly reduced.

Remarkably, before the initiation of systemic therapy, chest CT performed on June 5, 2023, revealed an extracranial abscopal effect, with regression of extracranial pulmonary lesions and metastatic lymph nodes exceeding 20% relative to baseline (**Figure 2**, **Supplementary Table S1**). Similarly, neurological symptoms, including right hemiplegia, headache, and projectile vomiting, showed significant improvement.

**Figure 3:**
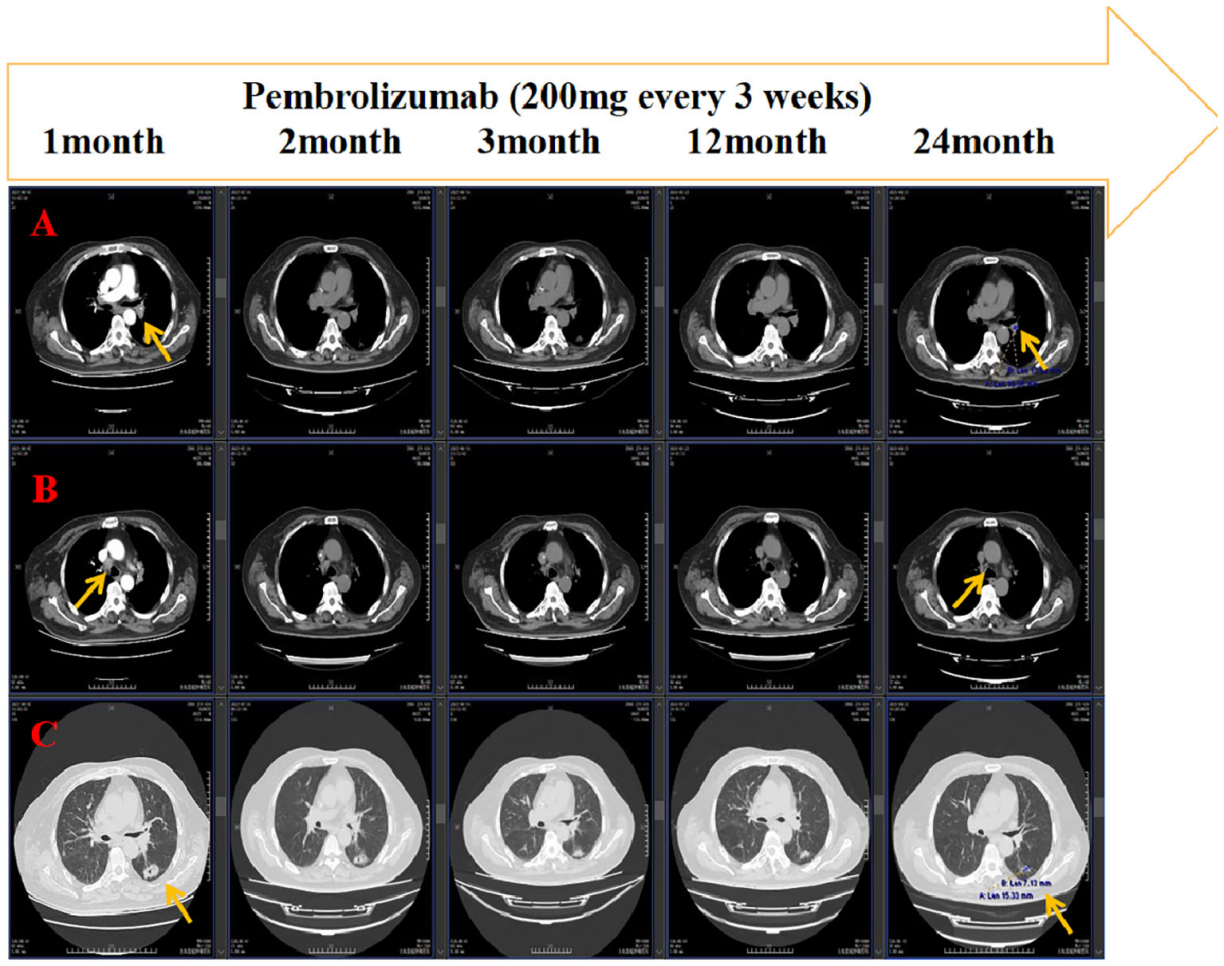
After treatment with pembrolizumab, Chest corkuted tomography [the left hilum of lung **(A)**, the right mediastinal lymph node **(B)** and the left lung lesion **(C)**] indicated sustained partial response of the extracranial lung lesions.

**Figure 4:**
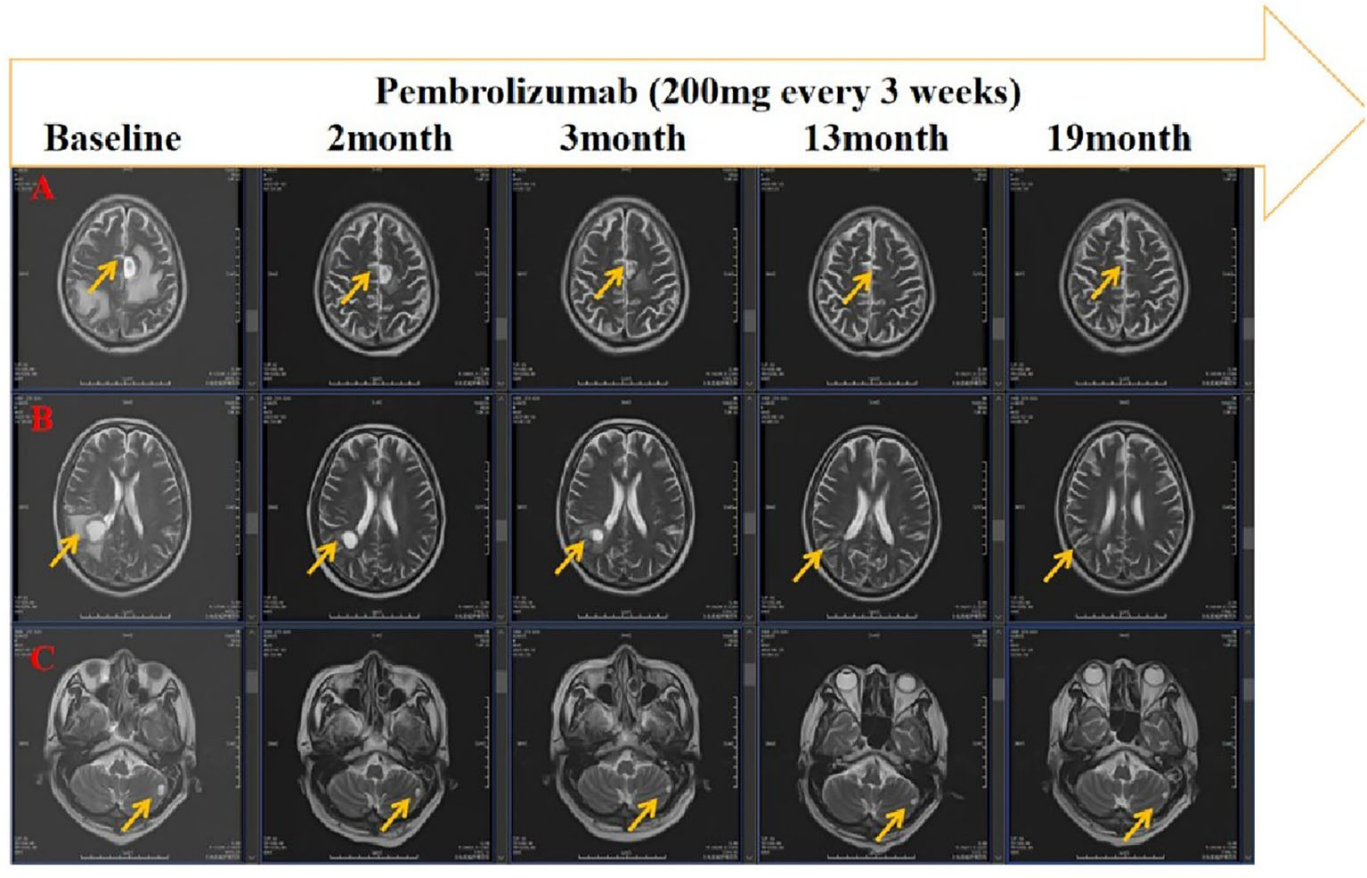
After treatment, head magnetic resonance imaging(MRI) indicated sustained partial response of the left frontal lobe **(A)**. right lateral ventricle posterior horn **(B)** and left cerebellum **(C)** lesions.

The supraclavicular lymph node specimen was subsequently submitted for genetic sequencing, which identified a KRAS G12C mutation, while EGFR, ALK, and ROS1 mutations were absent. Based on the molecular and immunohistochemical findings (PD-L1 TPS = 60%), pembrolizumab monotherapy was recommended in accordance with NCCN guidelines. Treatment with pembrolizumab (200 mg every 3 weeks) commenced in June 2023 and was continued until June 2025. The patient achieved a durable partial response both intracranially and extracranially, sustained for up to 24 months (**Figures 3**, **4**). During the course of pembrolizumab therapy, serum tumor markers carcinoembryonic antigen (CEA) and cytokeratin 19 fragment (CYFRA21-1) demonstrated a mild decline (**Supplementary Figure S2**) (**Supplementary Figure S3**).

## 3 Discussion

In this report, we describe a patient with advanced primary lung adenocarcinoma harboring a KRAS G12C mutation and high PD-L1 expression (TPS ≥50%) who underwent palliative brain radiotherapy followed by PD-1 antibody therapy. The patient achieved a durable partial response (PR) lasting over 24 months, experienced an extracranial abscopal effect, and did not develop severe systemic adverse events. These findings suggest that PD-1 blockade may play a pivotal role in sustaining antitumor immunity.

The abscopal effect is generally believed to be mediated through immune system activation (8). Radiotherapy can induce immunogenic cell death, resulting in the release of tumor-associated antigens. These antigens are subsequently processed by antigen-presenting cells (APCs), which activate cytotoxic T lymphocytes (CTLs) and natural killer (NK) cells, capable of eradicating distant tumor cells (9, 10). However, the extracranial abscopal effect following brain-directed radiotherapy remains exceedingly rare. This rarity is thought to be linked to the restrictive nature of the blood–brain barrier, which both limits the release of tumor antigens into the periphery and restricts APC infiltration into the brain parenchyma (6). Studies have shown the radiation fractionation is related to the occurrence of abscopal effect. Hypofractionated Radiotherapy schedules, especially single high dose(>3 Gy), seem the most effective regimen for inducing an abscopal effect (11, 12). High PD-L1 expression in tumor cells has been shown to drive immune evasion by inducing apoptosis of activated T cells, suppressing T-cell proliferation, and inhibiting effector functions within the tumor microenvironment (13, 14). Despite these mechanisms of immune suppression, our report is the first to demonstrate that an extracranial abscopal effect can still be elicited by brain radiotherapy alone in a patient with lung adenocarcinoma and PD-L1 TPS ≥50%, even before systemic immunotherapy was initiated.

Pembrolizumab remains the standard of care for NSCLC with PD-L1 overexpression (TPS ≥50%), with reported median objective response rates, progression-free survival, and overall survival of 44.8%, 10.3 months, and 26.3 months, respectively (15). Patients with symptomatic brain metastases requiring immediate radiotherapy often show significantly reduced survival outcomes (16). In this case, radiotherapy likely initiated an endogenous antitumor immune response, characterized by increased activation of effector and memory T-cell subsets (Tcm and Tem). Subsequent administration of pembrolizumab appeared to sustain and amplify this response, enabling durable disease control and maintaining partial response for 24 months despite the presence of brain metastases.

In conclusion, this case demonstrates that even within an immunosuppressive tumor microenvironment characterized by high PD-L1 expression (TPS ≥50%), brain radiotherapy alone has the potential to induce an abscopal effect. Further prospective studies are warranted to validate and expand upon these preliminary observations.

## Patient perspective

I was admitted to the hospital for treatment due to right-sided hemiplegia and inability to take care of myself in daily life. After undergoing brain radiotherapy and systemic immunotherapy, my symptoms have improved significantly, and the adverse reactions of the treatment are tolerable.

## Data Availability

The original contributions presented in the study are included in the article/supplementary material. Further inquiries can be directed to the corresponding authors.

## References

[1] Rybarczyk-Kasiuchnicz A Ramlau R Stencel K . Treatment of brain metastases of non-small cell lung carcinoma. Int J Mol Sci. (2021) 22(2):593. doi: 10.3390/ijms22020593, PMID: 33435596 PMC7826874

[2] Lahiri A Maji A Potdar PD Singh N Parikh P Bisht B . Lung cancer immunotherapy: progress, pitfalls, and promises. Mol Cancer. (2023) 22(1):40. doi: 10.1186/s12943-023-01740-y, PMID: 36810079 PMC9942077

[3] Wang Y Chen R Wa Y Ding S Yang Y Liao J . Tumor immune microenvironment and immunotherapy in brain metastasis from non-small cell lung cancer. Front Immunol. (2022) 13. doi: 10.3389/fimmu.2022.829451, PMID: 35251014 PMC8891382

[4] Wang S Hu C Xie F Liu Y . Use of programmed death receptor-1 and/or programmed death ligand 1 inhibitors for the treatment of brain metastasis of lung cancer. OncoTargets Ther. (2020) 13:667–83. doi: 10.2147/OTT.S235714, PMID: 32158220 PMC6986404

[5] Goldberg SB Schalper KA Gettinger SN Mahajan A Herbst RS Chiang AC . Pembrolizumab for management of patients with NSCLC and brain metastases: long-term results and biomarker analysis from a non-randomised, open-label, phase 2 trial. Lancet Oncol. (2020) 21:655–63. doi: 10.1016/S1470-2045(20)30111-X, PMID: 32251621 PMC7380514

[6] Lin X Lu T Xie Z Qin Y Liu M Xie X . Extracranial abscopal effect induced by combining immunotherapy with brain radiotherapy in a patient with lung adenocarcinoma: A case report and literature review. Thorac Cancer. (2019) 10:1272–5. doi: 10.1111/1759-7714.13048, PMID: 30929314 PMC6500971

[7] Hamilton AJ Seid J Verdecchia K Chuba P . Abscopal effect after radiosurgery for solitary brain metastasis from non-small cell lung cancer. Cureus. (2018) 10(12):e3777. doi: 10.7759/cureus.3777, PMID: 30854265 PMC6395017

[8] Demaria S Ng B Devitt ML Babb JS Kawashima N Liebes L . Ionizing radiation inhibition of distant untreated tumors (abscopal effect) is immune mediated. Int J Radiat OncologyBiologyPhysics. (2004) 58:862–70. doi: 10.1016/j.ijrobp.2003.09.012, PMID: 14967443

[9] Golden EB Frances D Pellicciotta I Demaria S Helen Barcellos-Hoff M Formenti SC . Radiation fosters dose-dependent and chemotherapy-induced immunogenic cell death. OncoImmunology. (2014) 3:e28518. doi: 10.4161/onci.28518, PMID: 25071979 PMC4106151

[10] Liu Y Dong Y Kong L Shi F Zhu H Yu J . Abscopal effect of radiotherapy combined with immune checkpoint inhibitors. J Hematol Oncol. (2018) 11(1):104. doi: 10.1186/s13045-018-0647-8, PMID: 30115069 PMC6097415

[11] Ghaffari-Nazari H Alimohammadi M Alimohammadi R Rostami E Bakhshandeh M Webster J T . Radiation dose and schedule influence the abscopal effect in a bilateral murine CT26 tumor model. Int Immunopharmacol. (2022) 108:108737. doi: 10.1016/j.intimp.2022.108737, PMID: 35417831

[12] Rocchetti JR Price J . Abscopal effect in a patient with metastatic melanoma receiving hypofractionated radiation therapy and dual immune checkpoint inhibition: A case report. Cureus. (2024) 16:e74662. doi: 10.7759/cureus.74662, PMID: 39735011 PMC11681968

[13] Yi M Zheng X Niu M Zhu S Ge H Wu K . Combination strategies with PD-1/PD-L1 blockade: current advances and future directions. Mol Cancer. (2022) 21(1):28. doi: 10.1186/s12943-021-01489-2, PMID: 35062949 PMC8780712

[14] Tang Q Chen Y Li X Long S Shi Y Yu Y . The role of PD-1/PD-L1 and application of immune-checkpoint inhibitors in human cancers. Front Immunol. (2022) 13. doi: 10.3389/fimmu.2022.964442, PMID: 36177034 PMC9513184

[15] Reck M Rodriguez-Abreu D Robinson GA Hui R Csőszi T Fülöp A . Five-year outcomes with pembrolizumab versus chemotherapy for metastatic non-small-cell lung cancer with PD-L1 tumor proportion score ≥ 50. J Clin Oncol. (2021) 39:2339–49. doi: 10.1200/JCO.21.00174, PMID: 33872070 PMC8280089

[16] Frost N Kollmeier J Misch D Vollbrecht C Grah C Matthes B . Pembrolizumab as first-line palliative therapy in PD-L1 overexpressing (≥ 50%) NSCLC: real-world results with special focus on PS ≥ 2, brain metastases, and steroids. Clin Lung Cancer. (2021) 22:411–22. doi: 10.1016/j.cllc.2021.02.001, PMID: 33648877

