# Supplementary figures and images for: Remarkable response to pembrolizumab in PD-L1 overexpressing (≥ 50%) NSCLC and extracranial abscopal effect induced by brain radiotherapy: a case report
Source: Front Oncol. 2025 Dec 12;15:1652515. doi: 10.3389/fonc.2025.1652515 (PMC12740888; doi:10.3389/fonc.2025.1652515)

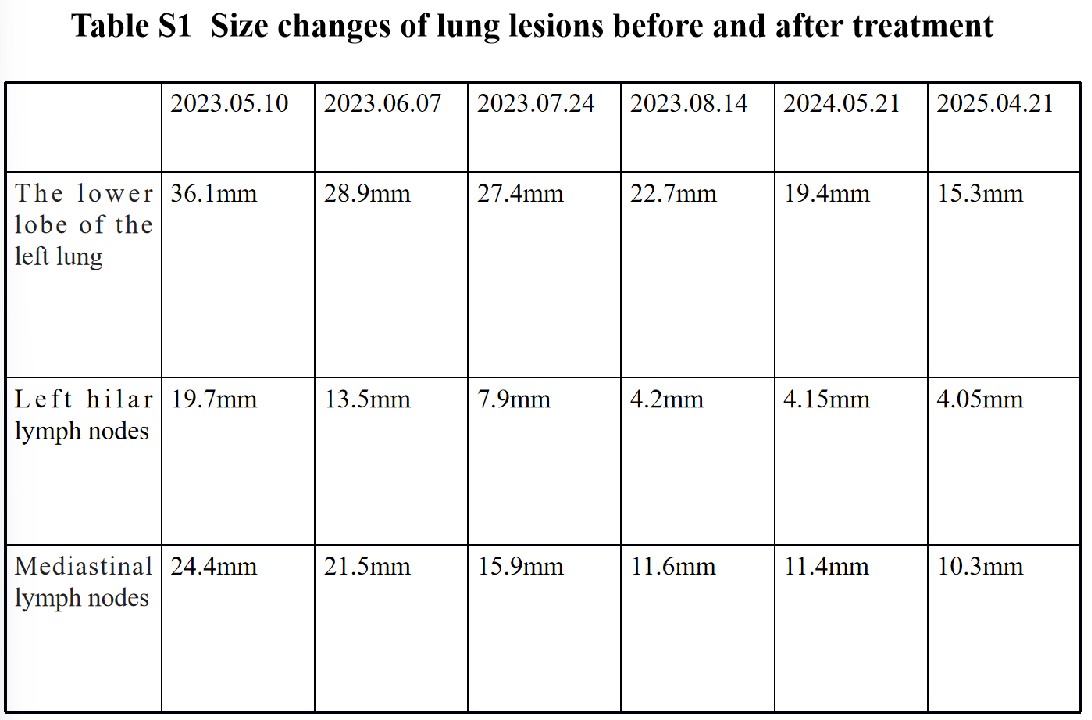

Supplement: Supplementary file 1 [file Table1.docx]

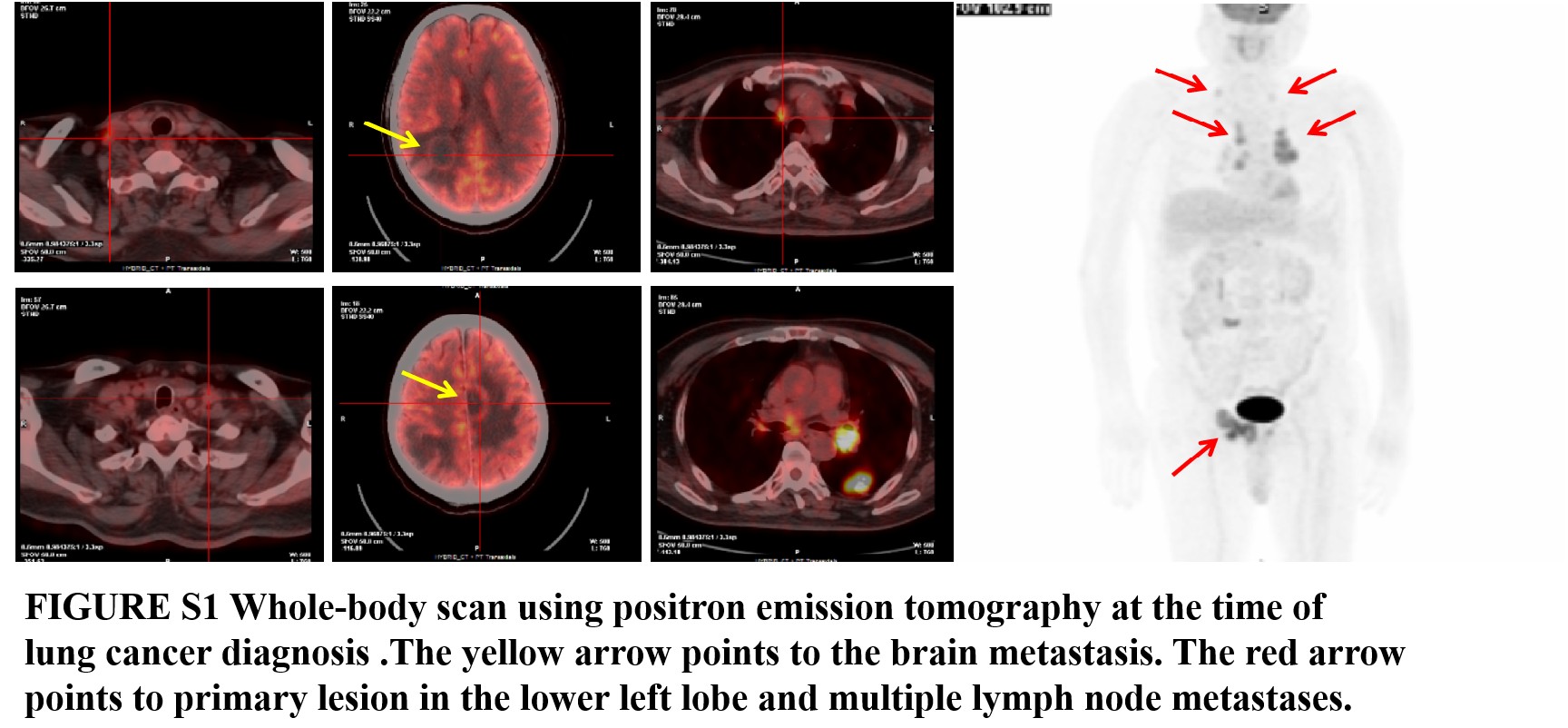

Supplement: Supplementary file 2 [file Image1.jpeg]

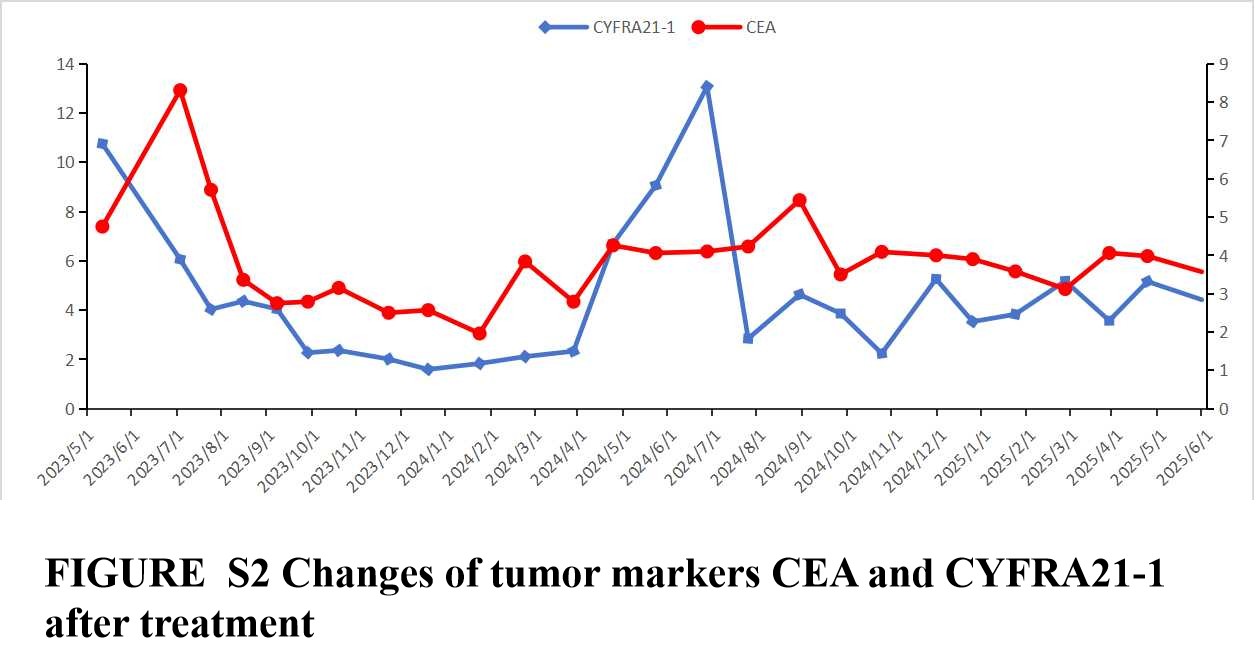

Supplement: Supplementary file 3 [file Image2.jpeg]

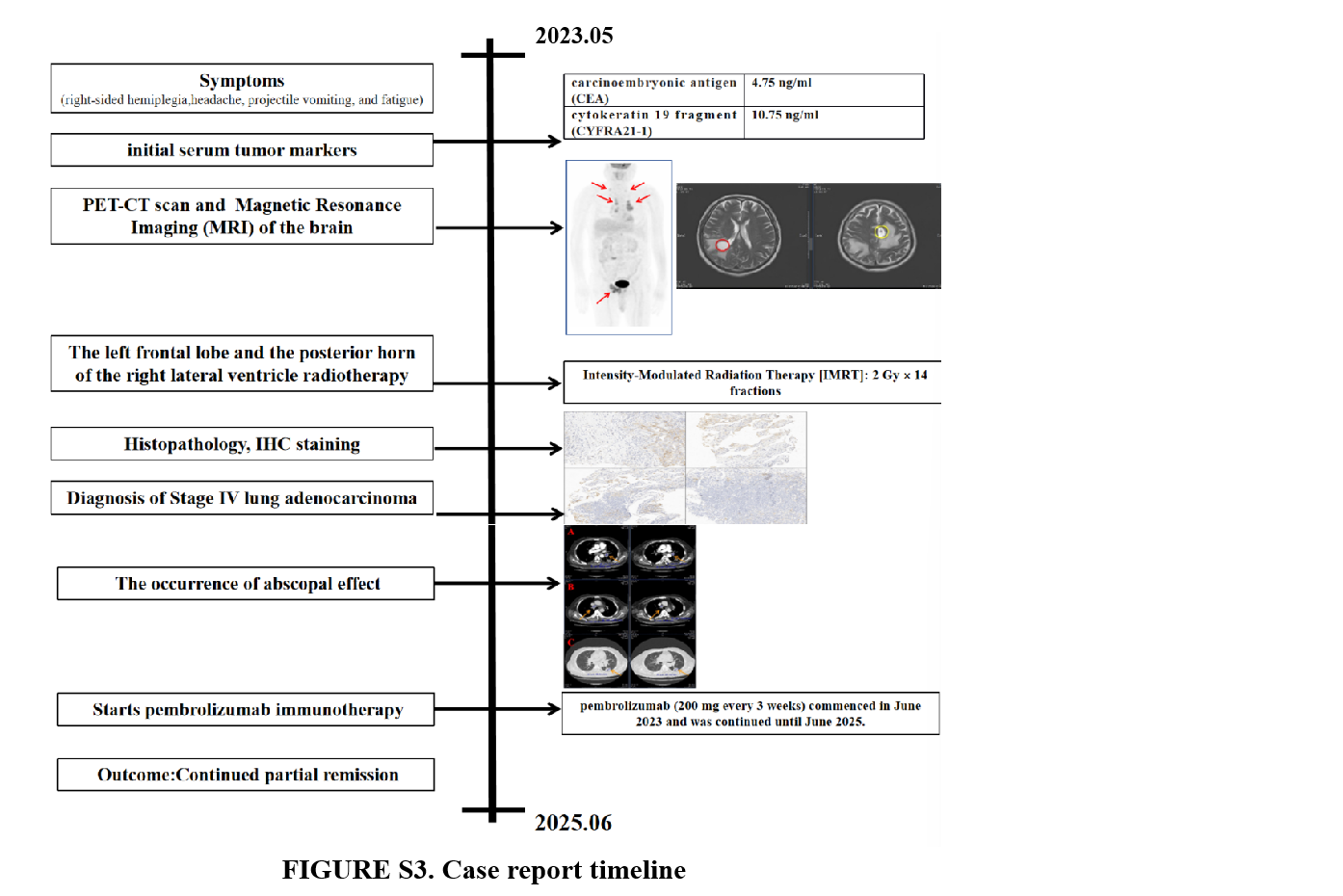

Supplement: Supplementary file 4 [file Image3.png]
